# *Medicago truncatula *transporter database: a comprehensive database resource for *M. truncatula *transporters

**DOI:** 10.1186/1471-2164-13-60

**Published:** 2012-02-06

**Authors:** Zhenyan Miao, Daofeng Li, Zhenhai Zhang, Jiangli Dong, Zhen Su, Tao Wang

**Affiliations:** 1 State Key Laboratory of Agrobiotechnology, College of Biological Sciences, China Agricultural University, Beijing, 100193, China; 2 State Key Laboratory of Plant Physiology and Biochemistry, College of Biological Sciences, China Agricultural University, Beijing, 100193, China

## Abstract

**Background:**

*Medicago truncatula *has been chosen as a model species for genomic studies. It is closely related to an important legume, alfalfa. Transporters are a large group of membrane-spanning proteins. They deliver essential nutrients, eject waste products, and assist the cell in sensing environmental conditions by forming a complex system of pumps and channels. Although studies have effectively characterized individual *M. truncatula *transporters in several databases, until now there has been no available systematic database that includes all transporters in *M. truncatula*.

**Description:**

The *M. truncatula *transporter database (MTDB) contains comprehensive information on the transporters in *M. truncatula*. Based on the TransportTP method, we have presented a novel prediction pipeline. A total of 3,665 putative transporters have been annotated based on International Medicago Genome Annotated Group (IMGAG) V3.5 V3 and the *M. truncatula *Gene Index (MTGI) V10.0 releases and assigned to 162 families according to the transporter classification system. These families were further classified into seven types according to their transport mode and energy coupling mechanism. Extensive annotations referring to each protein were generated, including basic protein function, expressed sequence tag (EST) mapping, genome locus, three-dimensional template prediction, transmembrane segment, and domain annotation. A chromosome distribution map and text-based Basic Local Alignment Search Tools were also created. In addition, we have provided a way to explore the expression of putative *M. truncatula *transporter genes under stress treatments.

**Conclusions:**

In summary, the MTDB enables the exploration and comparative analysis of putative transporters in *M. truncatula*. A user-friendly web interface and regular updates make MTDB valuable to researchers in related fields. The MTDB is freely available now to all users at http://bioinformatics.cau.edu.cn/MtTransporter/.

## Background

*Medicago truncatula *is closely related to an important forage legume, alfalfa. Because of its advantageous characteristics such as small size, short generation time, self-fertility, and diploid genome, *M. truncatula *has been used as a model species in genomic studies [1,2]. *Arabidopsis thaliana *is a model plant whose genome was sequenced by an international consortium and is well annotated. Very high sequence identity exists between genes from *M. truncatula *and their counterparts from alfalfa (98.7% at the amino acid level for isoflavone reductase and 99.1% at the amino acid level for vestitone reductase), so it serves as a genetically tractable model for alfalfa, which is tetraploid. In addition to alfalfa, *M. truncatula *can act as a model organism for economically important legumes such as soybeans [3]. Second only to the grass family, the legume family is important to humans as a source of food, feed for livestock, and raw materials for industry [4]. In a symbiotic association with rhizobia, legumes supply their own nitrogen by reducing N_2 _to NH_3_. This mutually beneficial association supplies a free and renewable source of available nitrogen for legumes and other crops [5]. By establishing symbiosis with mycorrhizal fungi, legumes also help the plant obtain phosphorous and other nutrients from the soil [6].

Transporters represent a large and diverse group of membrane-spanning proteins. They deliver essential nutrients, eject waste products, and assist the cell in sensing environmental conditions by forming a complex system of pumps and channels. Differences in membrane topology, energy-coupling mechanisms, and substrate specificities are present. Numerous studies have demonstrated that transporters play indispensable roles in the fundamental cellular processes of all organisms [7]. In addition, transporters provide pathogenic bacteria with resistance to antibiotics and provide cancer cells with resistance to chemotherapies. Systematic studies have been performed to identify and characterize the transporters in a variety of plant species, such as *Arabidopsis *and rice. With the assistance of databases containing known and characterized transport proteins, transporters in new species are identifiable and classified via sequence similarity. Perhaps the most comprehensive of these databases is the Transporter Classification Database (TCDB), which contains a large group of functionally characterized transporters. It also achieves the purpose of categorizing new transporters into families and subfamilies based on molecular, evolutionary, and functional properties [8,9].

However, although studies have characterized individual *M. truncatula *transporters in several databases, there has been no systematic database that includes all transporters in *M. truncatula*. Extensive cDNA and genomic DNA sequencing of several legume species (e.g., *M. truncatula*, soybeans, and *Lotus japonicas*) have been implemented over the past few years and have enabled an interesting model system to analyze whole-genome transporters [10-13]. The genomic sequence of *M. truncatula *is being annotated by the International Medicago Genome Annotated Group (IMGAG)[14], which described 47,529 genes in its version 3.5v3 of the genome sequence http://www.medicagohapmap.org/downloads_genome/Mt3.5/. Additional resources relevant to *Medicago *functional genomics include the *Medicago *genome portal at the Noble Foundation [15], which provides final annotation analysis results on *Medicago *genes. To help researchers interested in *M. truncatula *transport proteins, we report the development of the *M. truncatula *transporter database (MTDB), which contains information about *M. truncatula *transporters derived from a comparison to the protein sequences of TCDB and *A. thaliana*, the most well-studied genetic model plant. This archives 3,665 putative *M. truncatula *transport proteins belonging to 162 families. This represents 7.5% of all predicted proteins in *Medicago *and is in line with what has been found in other plant species. For example, transporter genes account for 4.6% of all *Arabidopsis *genes and 5% of all rice genes [16,17]. The aim of the MTDB is to present the comprehensive transporter profiles of sequenced *M. truncatula*, as well as to provide comparative and phylogenetic trees to view, search, and compare the transporter data in an easy-to-navigate format.

## Construction and content

### Genome sequence data acquisition

Protein sequences of *M. truncatula *and their annotations were derived from the IMGAG. Transport protein sequences of *A. thaliana *and their annotations came from TransportDB [18]. Our transporter data were downloaded from the TCDB web site in March 2011 and contained 6,068 transporters. Pfam annotations came from the Pfam database, version 24.0[19]. Three-dimensional (3D) structure annotations were provided by the Protein Data Bank (PDB)[20]. *Medicago *transporter annotations based on the IMGAG V3.5 V3 were derived from the *Medicago *genome portal at the Noble Foundation.

### Identification of putative transporters

**Figure 1:**
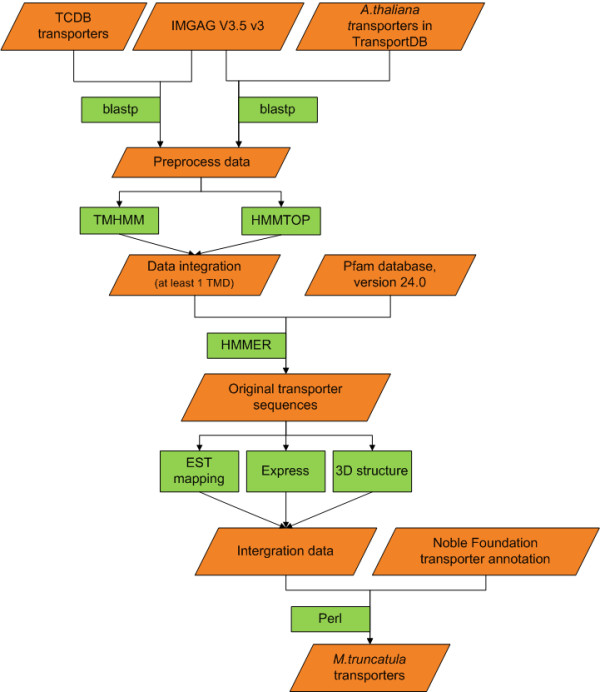
**Computational prediction to identify *Medicago truncatula *transporters**. We used Basic Local Alignment Search Tool (BLAST) and HMMER searches in computational predictions to identify *M. truncatula *transporters. First, we used the transport protein sequences of *Arabidopsis thaliana *and the Transporter Classification Database (TCDB) to conduct a BLAST search with *M. truncatula *protein sequences, provided by the International Medicago Genome Annotated Group (IMGAG). The preprocess data were then analyzed for the presence of a potential transmembrane domain (TMD) using two algorithms: TMHMM and HMMTOP 2.0. Afterward, we used the annotated sequences to conduct a HMMER search with the Pfam annotations from the Pfam database, version 24.0. In addition, all original transporters were compared with proteins of the *M. truncatula *transporter annotation at the Noble Foundation. We used Perl scripts to analyze the results.

In MTDB, we used Basic Local Alignment Search Tool (BLAST)[21] and HMMER [22] searches in computational predictions to identify putative *M. truncatula *transporters [Figure 1]. First, we respectively used 1,278 transport protein sequences of *A. thaliana *from TransportDB and 6,080 transporters in TCDB to conduct a BLASTp search with 47,529 *M. truncatula *protein sequences provided by the IMGAG. Of the 6,080 transporters in TCDB, 248 were transport proteins of *A. thaliana*, of which 181 were also found in TransportDB. We set the e-value cut-off at 0.0001 and identity at > 30% when we used Perl http://www.perl.org and BioPerl [23] scripts to analyze the BLASTp search results. A total of 5,706 (12.0%) *M. truncatula *proteins were predicted by at least one procedure, of which 1,974 were identified by two procedures. We selected only the top five homologs for easily storage. The 47,529 *M. truncatula *proteins were then predicted from the genome sequence (IMGAG sequence release version 3.5v3) and analyzed for the presence of a potential transmembrane domain (TMD) using two algorithms: TMHMM [24] and HMMTOP 2.0[25]. Of the IMGAG-annotated proteins, 17,471 (36.8%) were predicted by one or more programs to contain at least one TMD, of which 8,889 were identified by the two programs. In addition, we used the annotated sequences to conduct a HMMER search with the Pfam annotations that came from the Pfam database, version 24.0. We used Perl scripts to analyze the HMMER search result to obtain all annotated sequences whose pfamID were contained by the TCDB transporters and *A. thaliana *pfamID sets. In the end, 3,598 (7.5%) putative transport proteins that contained at least one TMD were annotated; they had sequence homology with proteins in TCDB and *A. thaliana *transporters. We also used SOSUI [26] web-based software to re-predict the protein transmembrane segment. Of the 3,598 putative transporters, 2,780 were predicted to contain at least one TMD (77.3%). Benedito et al. published a comprehensive analysis of *M. truncatula *transporters [8]. We compared our analysis with Benedito et al.'s published results, which were based on the IMGAG V2.0 (2,582). We mapped between transporters based on IMGAG V3.5v3 and IMGAG V2. Of the 3,598 putative transporters, 2,507 were assigned to 2,047 published transporters; the overlap rate was 79.3% and the validated rate was 69.7%. In addition, all 3,598 proteins were compared with proteins of the *Medicago *transporter annotation obtained from the Noble Foundation based on the latest IMGAG V3.5 V3. Of the 3,598 predicted transport proteins, 2,622 (72.9%) were also found in the *Medicago *transporter annotation at the Noble Foundation. Furthermore, we searched the annotation of the transporters based on IMGAG V3.5v3 occurring in the bioinformatics lab at the Noble Foundation but absent in our predictions using the keywords "transporter" and "transport." Finally, an additional 67 proteins were predicted.

We also mapped *Medicago *EST data (*M. truncatula *Gene Index [MTGI] version 10.0)[27] onto the 3,598 putative transport proteins using mutual BLASTn. We set the e-value cut-off at 10^-30 ^when we used Perl scripts to analyze the BLASTn search results. Of the 68,848 Tentative Consensus (TC) sequences and singletons in the EST database, 6,623 encoded proteins similar to our putative transport proteins.

In total, 3,665 putative transporters were annotated and assigned to 162 families according to the transporters in the TC classification system. These families were further classified into seven types according to their transport mode and energy coupling mechanism [Table 1]. All matching information was imported to the MTDB database to facilitate web searches and displays.

**Table 1:** Transport proteins in *Medicago truncatula *transporter database (MTDB) and classification according to Transporter Classification Database (TCDB) classes.

Type	Family	**MT number**^**a**^	**ARATH number**^**b**^	**TCDB number**^**c**^
1. Channels/pores	38	1073	150	1262
2. Electrochemical potential-driven transporters	49	1044	782	1643
3. Primary active transporters	22	1037	293	2211
4. Group translocators	3	25	0	139
5. Transmembrane electron carriers	6	75	0	109
8. Accessory factors involved in transport	11	81	0	148
9. Incompletely characterized transport systems	33	330	53	452

Total	162	3665	1278	5964

^a ^MT number: The number of putative *Medicago truncatula *transporters.

^b ^ARATH number: The number of transport proteins of *Arabidopsis thaliana *came from the TransportDB.

^c ^TCDB number: The number of transport proteins came from the TCDB web site in March 2011.

### Database architecture

We constructed and configured MTDB upon a typical LAMP (linux + Apache + MySQL + PHP) platform. The data set was stored in MySQL 4.1 http://www.mysql.com and a web interface was achieved using PHP scripts (PHP version 4.4; http://www.php.net) on Red Hat Linux, powered by an Apache sever. Schema of this database consists of five tables of the current version of MTDB [Additional file 1]. Table *pro *stores whole genome transport protein predictions and expressed sequence tag (EST) mapping data; table *domain *stores data related to the protein domain annotation predictions by Pfam; table *tmhmm *stores data related to transmembrane segment prediction by TMHMM; and table *structure *stores the experimentally determined 3D structures of membrane transporters. An additional table, *express*, stores information on the expression of putative *M. truncatula *transporter genes under stress treatments.

## Utility and discussion

### Web functions

**Figure 2:**
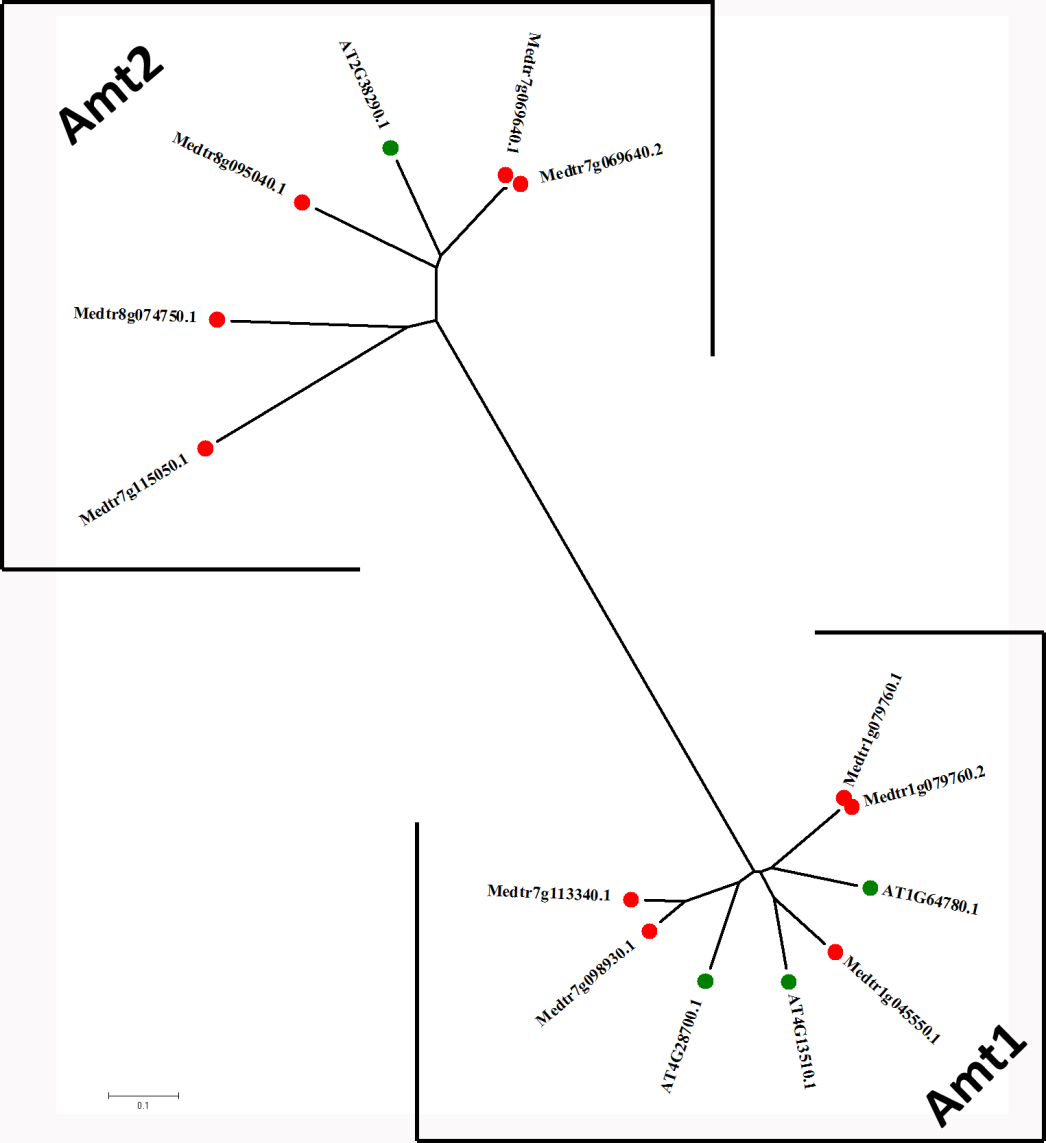
**Phylogenetic tree analysis of the Amt family**. This figure shows the result of phylogenetic tree analysis of Amt, an example of the 162 families in the *Medicago truncatula *transporter database. Individual members of the Amt family were further clustered and color-coded based on the result of phylogenetic analysis and TC numbering system. *M. truncatula *Amts can be classified into two groups: Amt1 and Amt2. Green markers refer to *Arabidopsis thaliana *sequences. Red markers refer to *M. truncatula *sequences. Each group contains links to individual protein pages.

**Figure 3:**
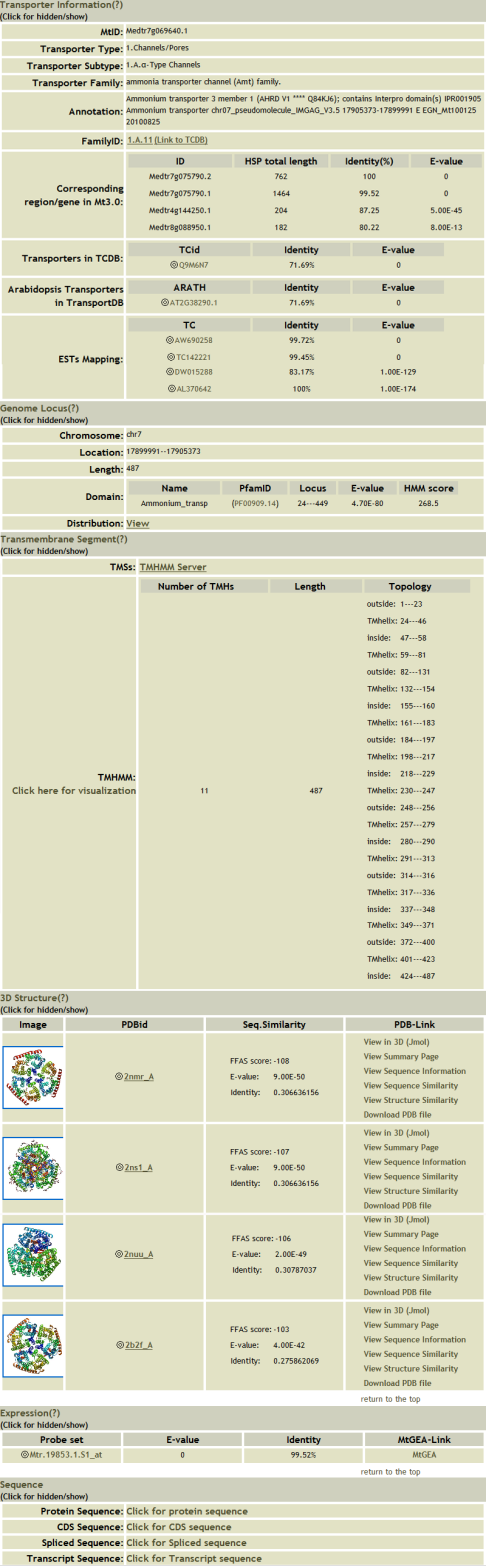
**Individual protein page**. Here we use a protein called "Medtr7g069640.1" from the Amt family as an example. This figure shows separate web pages where users can find detailed information such as transporter function annotation, transmembrane segment prediction by TMHMM, genomic locus information, expressed sequence tag mapping results, domain annotation prediction by Pfam, three-dimensional structure prediction, expression annotation, and protein/cDNA/spliced/transcript sequences.

**Figure 4:**
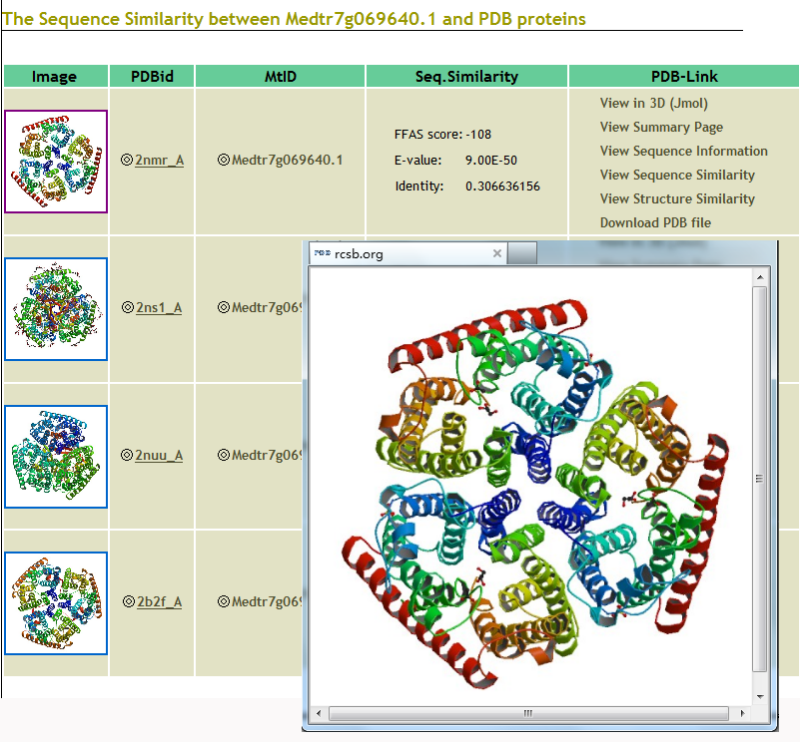
**Three-dimensional templates of *Medicago truncatula *transporters**. Here we use a protein called "Medtr7g069640.1" from the Amt family as an example. We obtained the reference sequences from the protein data bank (PDB). There are 10 members in Amt family, all of which pick up four consensus templates named 2NMR (structure of the *E. coli *Amt), 2NS1 (structure of the *E. coli *Amt), 2B2F (structure of the *A. fulgidus *Amt) and 2NUU (structure of the *E. coli *Amt). Links to PDB and the *M. truncatula *transporter database are also provided. The result is graphic.

We designed a user-friendly web site interface. Users can browse or search different functions of content classes based on various choices. Using the search function, for example, users can search for one type of putative transporter or specify certain information such as gene family, transporter name, or *M. truncatula *ID (MtID). In addition, a batch search function is achieved, through which users can search the transporter information by inputting a set of MtIDs. A brief summary on the page can provide users with a useful platform for searching. The MTDB supports a comprehensive treeview-like navigation interface. Users can browse the TC numbering system of every type of *M. truncatula *transporter with a collapsible function. The results are grouped by transporter family. To make browsing more convenient and scientific, we performed phylogenetic tree analysis of each family. Protein sequences in each family were used to generate a midpoint-rooted neighbor-joining tree. The trees were created by Mega 4.0[28] with the default parameters. Individual members of the families were further clustered into groups based on TC numbering system and evolutionary analysis [Figure 2 and Additional file 2]. Each group contains links to individual protein pages. Each putative transport protein is presented on separate web pages where users can find detailed information such as transporter function annotations; TC classifications; transmembrane segment predictions by TMHMM; genomic locus information; EST mapping results; domain annotation predictions by Pfam; 3D template predictions; expression annotations; and protein, cDNA, and genome sequences [Figure 3]. The protein and CDS sequences in MTDB are readily available for BLAST searches. Users can submit a single peptide or nucleotide sequence in the "BLAST" section. Location distribution maps, expression annotations, and 3D templates can also be browsed quickly using the "Advanced Tools" function [Figure 4].

### Comparative tools and references

A map containing gene loci located on the chromosomes was generated and visualized using GenomePixelizer [29], which gives users a direct view of the distribution of *M. truncatula*'s putative transporter genes on chromosomes and is especially useful in observing tandem duplications [Additional file 3]. The sections are included in the advanced tools function: structure, transmembrane segment, and expression. The "structure" section has been added to MTDB and describes putative transporters that have sequence homology with experimentally determined 3D structures. We used the transport protein sequences of *M. truncatula *to conduct a Position-Specific Iterative (PSI)-BLAST search with protein sequences provided by the PDB. We set the maximum number of iterations at three and the e-value cut off at 0.001 (PSI-BLAST-based method)[30]. In addition, we used the FFAS [31] tools to filter the (PSI)-BLAST results. Lower (more negative) FFAS scores indicate stronger similarity. FFAS scores lower than -9.5 are expected to contain less than 3% of false positives as indicated by comprehensive benchmarks of known structures. A total of 1,950 putative transporters were represented by structures in the PDB. Links to the PDB and MTDB are also provided.

In the "transmembrane segment" section, users can submit a single protein sequence to the service at http://www.cbs.dtu.dk/services/TMHMM/, and then TMHMM outputs statistics and a list of the locations of the predicted transmembrane helices and the predicted locations of the intervening loop regions. This information can be shown graphically.

### Mapping of probe sets onto transporter genes

The consensus sequence of each probe set was provided by Affymetrix [32]. A total of 3,665 predicted transporter coding sequences were matched to probe sets using mutual BLASTn. The annotation of the best match was assigned to the probe set (best BLAST hit method)[33]. We set the e-value cut-off as 10^-4 ^and the length of the high-scoring segment pair to be longer than 100 bp then we used Perl and BioPerl scripts to analyze the BLAST search results (Mapping method in MtED)[34]. In total, of the 3,665 putative transporters, 2,039 were represented by probe sets on the Affymetrix Medicago GeneChip. Probe sets mapping information for all identified transporters were imported into the MTDB database to facilitate web searches and displays.

### Microarray expression value

We further provided a way to explore the expression of putative *M. truncatula *transporter genes under stress treatments. To explore the expression of *M. truncatula *transporter genes, we retrieved and categorized microarray expression data from Gene Expression Omnibus (GEO)[35]. We picked up two independent GEO series, GSE13921 and GSE27991. GSE13921 was provided by MtED which includes functional category analysis, some querying and maps tools, and tools for the comparison and visualization of expression profiles. We mainly used MtED's data because of its high quality and experimental continuity. MtED collects roots at 0 h, 6 h, 24 h, and 48 h after salt stress for microarray experiments. The expression of probe sets at one time point changed more or less than two-fold versus 0 h and was described as up-regulated or down-regulated, respectively. Based on the result obtained from express information analysis, at 6-h stress, 47.7% of the transporter genes (972) were up-regulated and 52.2% of the transporter genes (1,064) were down-regulated. At 24-h stress, 49.6% of the transporter genes (1,011) were up-regulated and 50.4% of the transporter genes (1,028) were down-regulated. At 48-h stress, 50.3% of the transporter genes (1,026) were up-regulated and 49.5% of the transporter genes (1,009) were down-regulated. Besides, another GEO series, GSE27991, collects expression data of *M. truncatula *roots treated with auxin transport inhibitors. We made pairwise comparisons within each series grown under same condition respectively and users can directly inspect gene expression values by searching any one of the MtID. Each result contains links to the experiment page, which provides users with the expression curve graphs and other annotation links.

The *M. truncatula *Gene Expression Atlas (MtGEA)[36] is a comprehensive platform that provides complete transcription profiles of all major organ systems of *M. truncatula*. We included suitable links to MtGEA on the expression page and transporter detail page so that users can readily examine transcriptome information for their probe set of interest in the MTDB.

In the future, we will continue to incorporate new expression information. Regular update and relative analysis will provide user up-to-data transporter expression information.

## Future prospects

MTDB was developed as a relational database for the comprehensive representation of *M. truncatula *transporter systems. As the *M. truncatula *genome is currently being annotated by an international consortium, available information on this model legume (including sequences, 3D structures, expression and pathway information) will become more comprehensive and accurate. MTDB will be routinely updated monthly with new annotation information.

## Conclusion

In summary, we built a local database called MTDB that was constructed in the PHP scripting language as a MySQL relational database system based on a Linux server. The MTDB is the first convenient web-based index database concerning transporters in the model legume *M. truncatula*. It will assist searchers in related fields by providing comprehensive information on transporter gene families and members of these families. The MTDB enables the exploration and comparative analysis of putative transporters in *M. truncatula*. A total of 3,665 putative transport proteins have been annotated and assigned to 162 families according to the TC classification system. These families are further classified into seven types according to their transport mode and energy-coupling mechanism. Both manual management and automated searches were achieved for the identification of putative protein sequences. Extensive annotations referring to each protein were generated, including basic protein function, genome locus, sequence annotations, EST mapping results, 3D template predictions, transmembrane segments, and domain annotation. A chromosome distribution map and text-based and BLAST search tools against known sequences of *M. truncatula *were also created. A user-friendly web interface and regular updates make MTDB valuable to researchers in related fields. We further provided a way to explore expression of *M. truncatula *transporter genes under stress treatments. The MTDB is freely available now to all users at http://bioinformatics.cau.edu.cn/MtTransporter/.

## Availability and requirements

The database is available at http://bioinformatics.cau.edu.cn/MtTransporter/ and is usable with most modern web browsers. The user's browser must have JavaScript enabled to show query examples and Cookie and Flash to show the expression curves.

## Authors' contributions

ZM constructed the database and drafted the manuscript and DL provided assistance. ZZ provided web-server system support. JD, SZ, and WT supervised the project. All authors read and approved the final manuscript.

## Supplementary Material

Additional file 1 **MySQL database structure model for the *Medicago truncatula *transporter database**. We use MySQL 4.1 to store our data set. Click here for file

Additional file 2 **Phylogenetic tree analysis of the MIP family**. This figure shows the result of phylogenetic tree analysis of MIP, one example of the 162 families in the *Medicago truncatula *transporter database. Individual members of the MIP family were further clustered and color-coded based on the result of phylogenetic analysis and TC numbering system. As observed in *Arabidopsis, M. truncatula *MIPs also can be classified into four groups: NIP, SIP, PIP, and TIP. Green markers refer to *Arabidopsis thaliana *sequences. Red markers refer to *M. truncatula *sequences. Click here for file

Additional file 3 **Distribution map of transporters**. The distribution map presents the locations of transporter genes. Genes are represented by squares and color-coded according to their types. Clicking any block will redirect to the corresponding individual protein page. Click here for file
