# Supplementary figures and images for: Medicago truncatula transporter database: a comprehensive database resource for M. truncatula transporters
Source: BMC Genomics. 2012 Feb 6;13:60. doi: 10.1186/1471-2164-13-60 (PMC3298476; doi:10.1186/1471-2164-13-60)

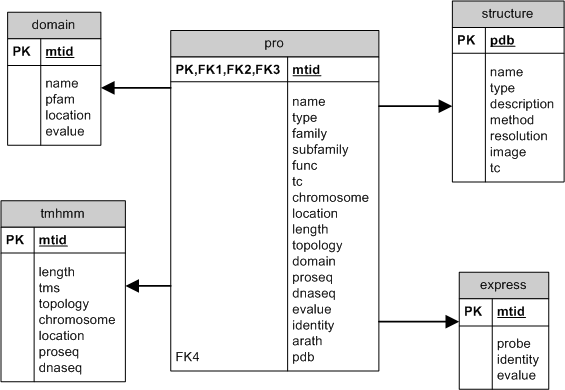

Supplement: Additional file 1 — MySQL database structure model for the Medicago truncatula transporter database. We use MySQL 4.1 to store our data set. [file 1471-2164-13-60-S1.PNG]

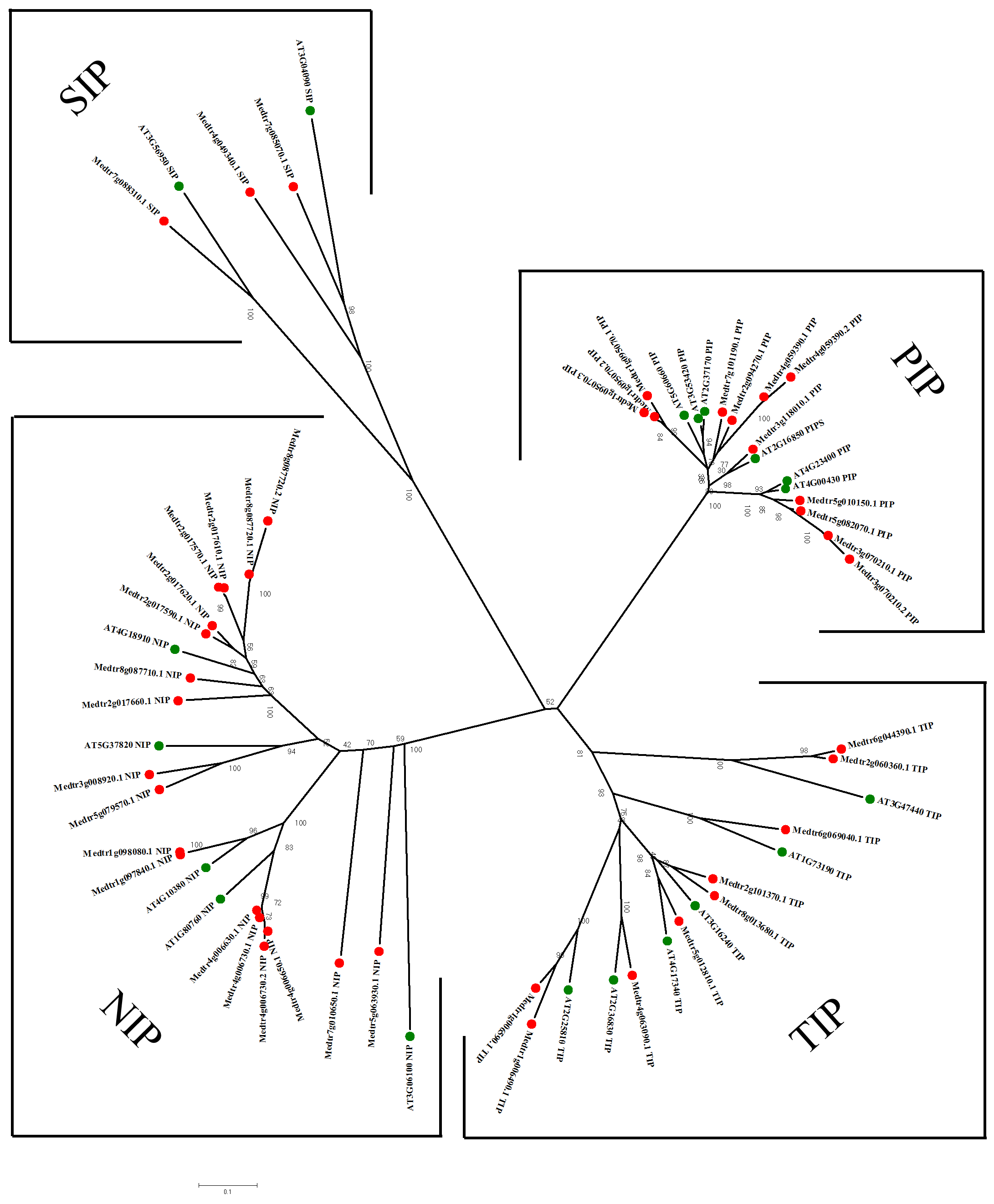

Supplement: Additional file 2 — Phylogenetic tree analysis of the MIP family. This figure shows the result of phylogenetic tree analysis of MIP, one example of the 162 families in the Medicago truncatula transporter database. Individual members of the MIP family were further clustered and color-coded based on the result of phylogenetic analysis and TC numbering system. As observed in Arabidopsis, M. truncatula MIPs also can be classified into four groups: NIP, SIP, PIP, and TIP. Green markers refer to Arabidopsis thaliana sequences. Red markers refer to M. truncatula sequences. [file 1471-2164-13-60-S2.TIFF]

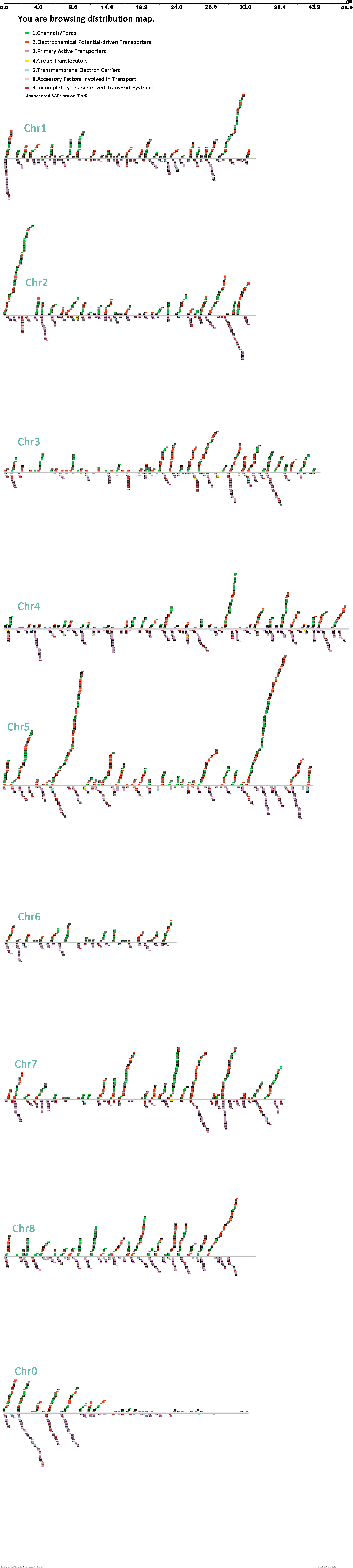

Supplement: Additional file 3 — Distribution map of transporters. The distribution map presents the locations of transporter genes. Genes are represented by squares and color-coded according to their types. Clicking any block will redirect to the corresponding individual protein page. [file 1471-2164-13-60-S3.JPEG]
